# Mechanical failure of distal femur mega prosthesis due to polyaryl-ether-ether-ketone (PEEK) hinge component

**DOI:** 10.1007/s00402-024-05306-5

**Published:** 2024-04-20

**Authors:** Omri Merose, Shai Factor, Yair Gortzak, Solomon Dadia, Ortal Segal, Aya Vituri, Arie Bussiba, Amir Sternheim

**Affiliations:** 1 Division of Orthopedic Surgery, Tel Aviv Medical Center, 6 Weizmann St, Tel Aviv, 6423906 Israel; 2 Affiliated to Faculty of Medicine, Tel Aviv University, Tel Aviv, Israel; 3 Tel Aviv University Center for AI and Data Science (TAD), Tel Aviv, Israel; 4 Department of Materials Engineering, Ben Gurion of the Negev, P.O.Box 653, Beer Sheva, Israel

**Keywords:** Polyaryl-ether-ether-ketone, PEEK, Biomechanical failure, Prostheses, Femur reconstruction

## Abstract

**Background:**

Polyaryl-ether-ether-ketone (PEEK) has gained popularity as a substrate for orthopaedic hardware due to its desirable properties such as heat and deformation resistance, low weight, and ease of manufacturing. However, we observed a relatively high failure rate of PEEK-based hinges in a distal femur reconstruction system. In this study, we aimed to evaluate the proportion of patients who experienced implant failure, analyse the mechanism of failure, and document the associated clinical findings.

**Methods:**

We conducted a retrospective cohort study, reviewing the medical charts of 56 patients who underwent distal femur resection and reconstruction with a PEEK Optima hinge-based prosthesis between 2004 and 2018. Concurrently, we performed a clinical and biomechanical failure analysis.

**Results:**

PEEK component failure occurred in 21 out of 56 patients (37.5%), with a mean time to failure of 63.2 months (range: 13–144 months, SD: 37.9). The survival distributions of PEEK hinges for males and females were significantly different (chi-square test, p-value = 0.005). Patient weight was also significantly associated with the hazard of failure (Wald’s test statistic, p-value = 0.031).

**Discussion:**

Our findings suggest that PEEK hinge failure in a distal femur reconstruction system is correlated with patient weight and male gender. Retrieval analysis revealed that failure was related to fretting and microscopic fractures due to cyclic loading, leading to instability and mechanical failure of the PEEK component in full extension. Further assessment of PEEK-based weight bearing articulating components against metal is warranted.

**Supplementary Information:**

The online version contains supplementary material available at 10.1007/s00402-024-05306-5.

## Introduction

Multiple challenges are associated with successful joint reconstruction following a distal femur resection. The loss of bone and soft tissue knee joint ligaments are significant mechanical issues that must be effectively addressed [1]. One potential solution is the use of specialized hinged tumor mega prostheses, which offer inherent joint stability, predictable and functional range of motion, and the ability for immediate weight bearing postoperatively [2, 3]. Modular distal femur reconstruction systems, offered by various manufacturers, provide a range of implant sizes and designs that allow for the accurate assembly of optimal size prostheses to replace both resected bone and soft tissue joint restraints [4]. This facilitates the planning and execution of tumor resection and reconstruction surgery in a manner similar to primary joint arthroplasty.

Modern modular fixed or rotating hinge knee systems have remained relatively unchanged in terms of their conceptual design over the past three decades. While these systems have shown acceptable durability and reliability compared to non-tumor knee arthroplasty systems, the search for more durable and better-performing prostheses continues. The current systems are mainly composed of a metal and polyethylene combination, and there is a need for innovations that can improve their performance and longevity [5, 6]. Mega-prosthesis revision surgery can be both physically and financially taxing for patients and the healthcare system. While peri-prosthetic joint infections are the most common cause of early revision surgery, aseptic loosening or structural implant failure often result in late revisions [4]. In their study of 669 patients undergoing fixed-hinge knee megaprosthesis for musculoskeletal tumors, Ruggieri et al. found higher survival rates to breakage with the Howmedica Modular Reconstruction System (HMRS®, Stryker, UK) compared to the Kotz Modular Femur-Tibia Reconstruction system (KMFTR®, ,Stryker, UK) prosthesis at both 10 and 20 years of follow-up, with statistically significant differences [7]. In 2014, Henderson et al. categorized endoprosthesis reconstruction failure following limb salvage surgery into three general categories, which include mechanical, non-mechanical, and pediatric [8]. These categories were further divided into six failure modes, namely soft tissue, aseptic loosening, structural, infection, tumor progression, and pediatric (type 1 to 6 respectively).

Pala et al. investigated several mega prostheses of the knee in tumor and revision surgeries and reported that despite advancements, complications remain common, with infection often cited as a leading cause of failure. Silver-coated implants and the use of the gastrocnemius muscle flap can mitigate infection risks, while noninvasive expandable prostheses are crucial in pediatric cases to prevent leg length discrepancies [9]. This study focuses on mechanical structural failure of a hinge component composed of Polyaryl-ether-ether-ketone (PEEK).

In the late 1980s, the medical industry recognized the biocompatibility of PEEK, a polyaromatic ketone polymer. PEEK possesses biological and physical traits that are closer to bone as compared to traditional metal and polyethylene implants, including relative heat and deformation resistance and a lower creep rate [10–13]. PEEK is lightweight and easy to manufacture, and its ability to withstand chemical and radiation damage, as well as its reduced distortion on three-dimensional imaging, has led to numerous studies exploring its potential as a substrate for orthopedic load-bearing implants [14]. While it has shown promise in studies focusing on its use in fracture fixation plates in orthopedic trauma [15–17], reports of PEEK’s failure as an articulating component against metallic counter face are gradually emerging [18, 19]. PEEK’s reduced structural stiffness may lead to reduced interface stresses, eventually resulting in deformation and instability of knee arthroplasty prostheses, as described by Abdullah et al. for PEEK femoral components [20, 21].

The MUTARS® (Modular Universal Tumor and Revision System by Implantcast in Buxtehude, Germany) system, introduced in 1992, is widely distributed worldwide. In 2003, a new PEEK-based locking mechanism was introduced for the system’s distal femur reconstruction, known as the MUTARS® PEEK Optima®, which is still produced upon request. However, in 2015, the company released the new MK generation of the MUTARS® system, featuring a metal femoral hinge and discontinued the routine use of the PEEK hinge system. PEEK-OPTIMA™ (Invibio Ltd, UK) has been considered as an alternative joint arthroplasty bearing material comparable to Cobalt Chrome, in the distal femur component due to its favorable mechanical properties and the biocompatibility of its wear debris [22]. In this situation the PEEK optima is coupled with an Ultra High Molecular Weight PolyEthylene (UHMWPE) tibial component.

This study addresses the mechanism of failure of the PEEK-Optima hinge, including analyzing the root cause for the failure, the proportion of patients who experienced structural prosthesis failure, and documenting the characteristic symptoms and clinical findings associated with it.

## Materials and methods

This study is a retrospective cohort analysis that involved a review of patient medical records in our institute, following approval from the ethics committee. The database was examined from January 2004 to October 2018, during which 56 patients in our tertiary medical center underwent distal femur resection and reconstruction utilizing the MUTARS PEEK Optima system. The procedure was performed to treat various bone pathologies, with osteosarcoma being the most common underlying diagnosis in 40 out of 56 patients. Our study began in 2004 when we initially started using the IMPLANTCAST’s MUTARS system with the PEEK Optima hinge, and it ended in 2016 with our last utilization of this system for a primary distal femur reconstruction procedure.

After undergoing reconstruction surgery on their distal femur, patients were scheduled for regular appointments at the outpatient clinic for follow-up. They were initially seen at 3-, 6-, and 12-week post-surgery, and every 3 months for the first two years. After the two-year mark, the time between appointments gradually increased. The radiologic follow-up for the operated extremity included full leg length plain X-rays every 3 months and full-length femur MRI’s every 6 months for the first two years, followed by yearly MRIs thereafter. Chest CT scans and X-rays were used for systemic follow-up. In this retrospective study, we evaluated X-ray imaging data to measure the tibial base plate angle and the length of the femoral component. These parameters were assessed to analyze their association with the hazard of PEEK lock failure.

During the clinical follow-up, the patient’s gait and leg length were evaluated, and the operated extremity was inspected and palpated. Next, the operated knee’s range of motion and stability were assessed. PEEK lock failure, which is characterized by obvious knee hyperextension, was detected through physical examination. However, in some cases, the clinical presentation differed and involved mediolateral instability with little or no hyperextension. A careful history may aid in diagnosing PEEK lock failure, as patients may report a specific moment when their knee became unstable and complain of instability while going downstairs. Although various imaging techniques were used, none were able to demonstrate the fracture of the PEEK lock.

### Biomechanical failure analysis

To investigate the root cause of PEEK mechanical failure, three failed PEEK lock components were analyzed using the following methods:

Visual inspection involved surveying the PEEK lock’s surface with a high-definition camera and stereographic microscope. This was performed to classify the macro-fractures modes, determine their relation to mechanical overloads, and to locate the origin of crack initiation. The lock was also examined for possible manufacturing defects, such as macroscopic damage and their location compared to an unused lock.

Fractographic examination was carried out using a scanning electron microscope (SEM) in order to classify micro-fracture modes. This was done after coating the fracture surface with gold. Since PEEK is non-conductive, the gold medium was used to reveal the fracture’s specific features, aid in understanding the material’s failure modes, and identify the initiation site of the fracture. Broken locks were analyzed to demonstrate plastic deformation and wear traces, while an intact lock was examined to track the wear process on its interaction surfaces with both the implant’s polyethylene and metal components.

Metallographic examination using optical microscopy involved performing a perpendicular metallographic cross-section in areas where wear traces were detected. This helped determine the depth and orientation of microcracks beneath the surface.

### Statistical methods and analysis

Kaplan-Meier survival analysis was used for categorical variables. Cox regression was used to ascertain the effects of a continuous variable or multiple variables upon the time of failure. P-values were corrected for multiple comparisons using the Benjamini–Hochberg (BH) procedure. *P* < 0.05 was considered as statistically significant. Statistical analyses were performed using IBM SPSS version 28 and R version 4.2.2. To identify potential predictors of failure hazard, we performed Kaplan-Meier survival analysis on gender, primary tumor vs. metastasis or complication, and anatomic location. We used log-rank tests to evaluate differences in survival distributions among the different groups within each variable. Data regarding the patient’s weight and consequently the BMI, was missing in 10. These observations were excluded from regressions on patients’ weight.

## Results

Table 1 presents the demographic and clinical characteristics of 56 patients who underwent distal femur resection and reconstruction using the MUTARS PEEK Optima system. The mean age of patients at the time of primary reconstructive surgery was 30 years (range: 7–86 years, SD: 21.46), with a male to female ratio of 35:21. Among them, 48 patients had bone sarcoma, with osteosarcoma being the most common (*n* = 40, 71.4%). Furthermore, chondrosarcoma was identified in 5 patients (8.9%), while metastatic carcinoma, metastatic myxoid liposarcoma, and multiple myeloma each accounted for 1 case (1.8%) respectively. The mean length of follow-up was 71.32 months (range: 2-244 months, SD: 61.2). Patients with less than a year of follow-up had unfortunately passed away within 1 year following the index procedure. Notably, none of these patients experienced a PEEK failure during the observed follow-up period. This outcome underscores the critical nature of the disease and the challenges associated with achieving long-term follow-up in patients with bone sarcomas. PEEK component failure occurred in 21 of the 56 patients (37.5%), with a mean time to failure of 63.19 months (range: 13–144 months, SD: 37.9). Among patients who experienced PEEK component failure, 14 underwent minor revision surgery, replacing only the damaged component, while 12 underwent complete prosthesis revision using the newer MUTARS MK (Modular Knee) system. These indications primarily include cases of implant loosening and infections necessitating the exchange of both the tibial and femoral components. After minor revision surgery, two patients experienced additional PEEK failures. One patient encountered the subsequent PEEK failure 6 months post-revision surgery, while the second patient experienced it after 36 months.

**Table 1:** Patient demographics

Age (years)	30 ± 21.5
Gender	
Male	35 (62.5%)
Female	21 (37.5%)
Bone Sarcoma (*n* = 48)	48 (86%)
Osteosarcoma (*n* = 40)	40 (71.4%)
Follow-up Duration (months)	71.3 ± 61.2
PEEK Component Failure	21 (37.5%)
Time to PEEK Component Failure (months)	63.2 ± 37.9
Weight at Time of PEEK Failure (kg)*	75.5 ± 15.7
BMI at Time of PEEK Failure*	24.9 ± 3.9
Time to Failure or End of Follow-up (months)	50.1 ± 44.6
Minor Revision Surgery	14
Complete Revision Surgery	12
Tibia Baseplate Angle (deg)	90 ± 1.1
Femoral Component Length (cm)	20.5 ± 9.3

Values are presented as mean ± standard deviation

*Data is missing for 10 patients

The PEEK survival distributions for males and females were found to be significantly different, with a chi-square test of 7.807 (p-value = 0.005). However, there was no significant difference observed between primary tumor and metastasis or complication, with a chi-square test of 0.039 (p-value = 0.844). The variables of tibial base plate angle, femoral component length, and age at surgery were found to have no significant association with the hazard of PEEK lock failure. Cox regressions were performed for tibia baseplate angle (Wald (1) = 0.192, p-value = 0.661), femoral component length (Wald (1) = 0.084, p-value = 0.773), and age at time of surgery (Wald (1) = 2.391, p-value = 0.122).

The analysis revealed a significant association between patient weight and the hazard of failure, with Wald’s test statistic yielding a value of 9.571 and a p-value of 0.002. Additionally, patient weight at the time of PEEK failure was also found to be significantly associated with the hazard of failure, with Wald’s test statistic yielding a value of 4.641 and a p-value of 0.031. Conversely, BMI (with a p-value just below 0.1) and BMI at the time of PEEK failure failed to reach statistical significance, with Wald’s test statistics of 3.667 and 3.032, respectively. Due to high correlation among these four variables, only patient weight at time of surgery was included in the multivariate Cox model.

A multivariate Cox regression analysis was conducted to examine the influence of gender and patient weight measured at the time of failure on the outcome. The interaction term was not statistically significant (Wald (1) = 0.422, *p* = 0.516) and was eliminated from the model. The covariates were not dependent on time (χ2(2) = 0.562, *p* = 0.755), indicating that the proportional hazards assumption was met. Results showed that females had a significantly lower hazard of failure compared to males (Wald (1) = 4.387, *p* = 0.036, HR = 0.242, CI: [0.064, 0.913]). Additionally, higher patient weight was found to be a significant positive predictor of failure hazard (Wald (1) = 7.733, *p* = 0.005, HR = 1.050, CI: [1.014, 1.087]).

## Biomechanical analysis results

**Fig. 1:**
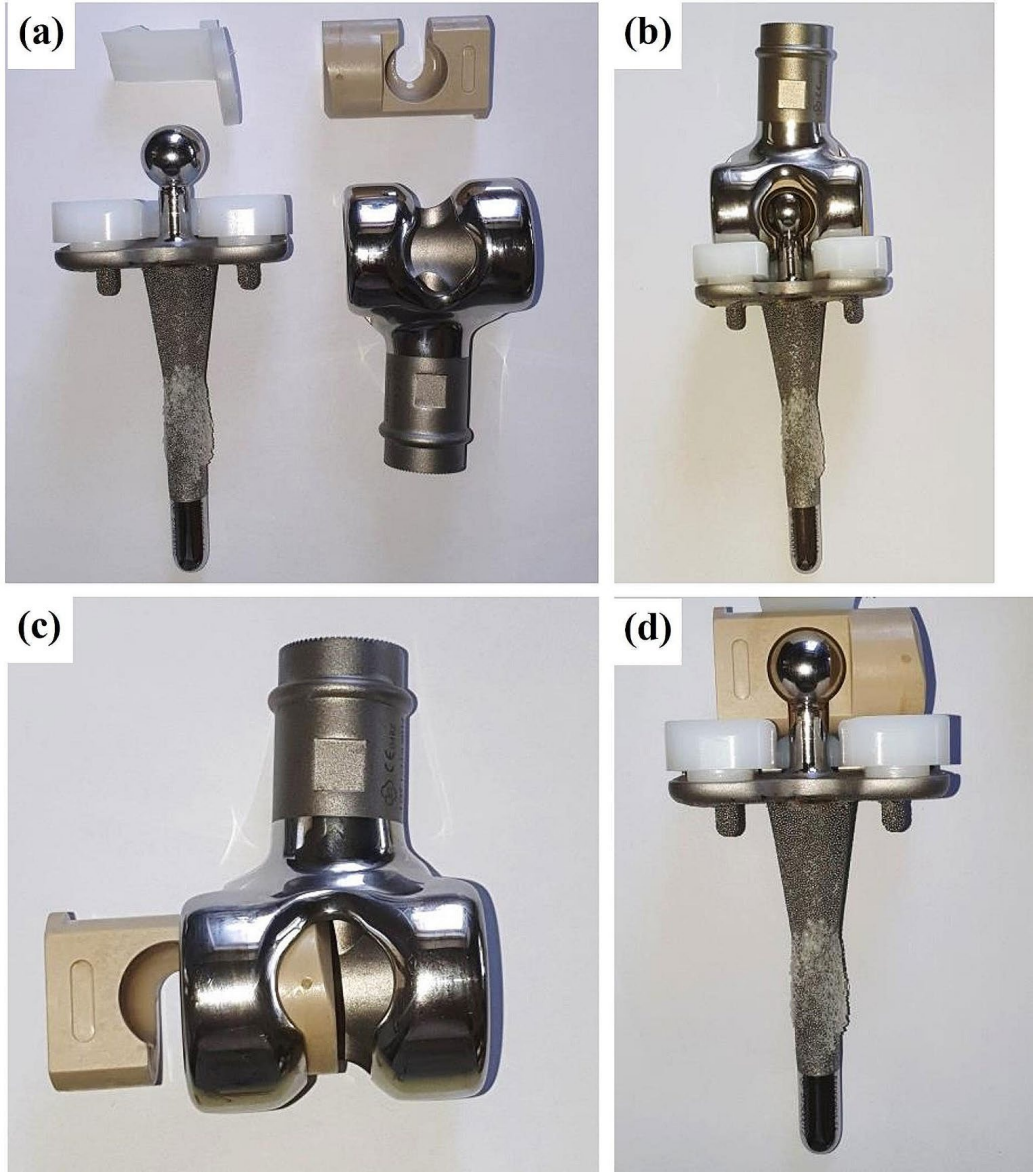
The components of the distal femur prosthesis; (**a**) general view, (**b**) the assembled design, (**c**) the PEEK hinge component within the metallic house of the distal femur component, (**d**) the tibial component within the PEEK hinge component

**Fig. 2:**
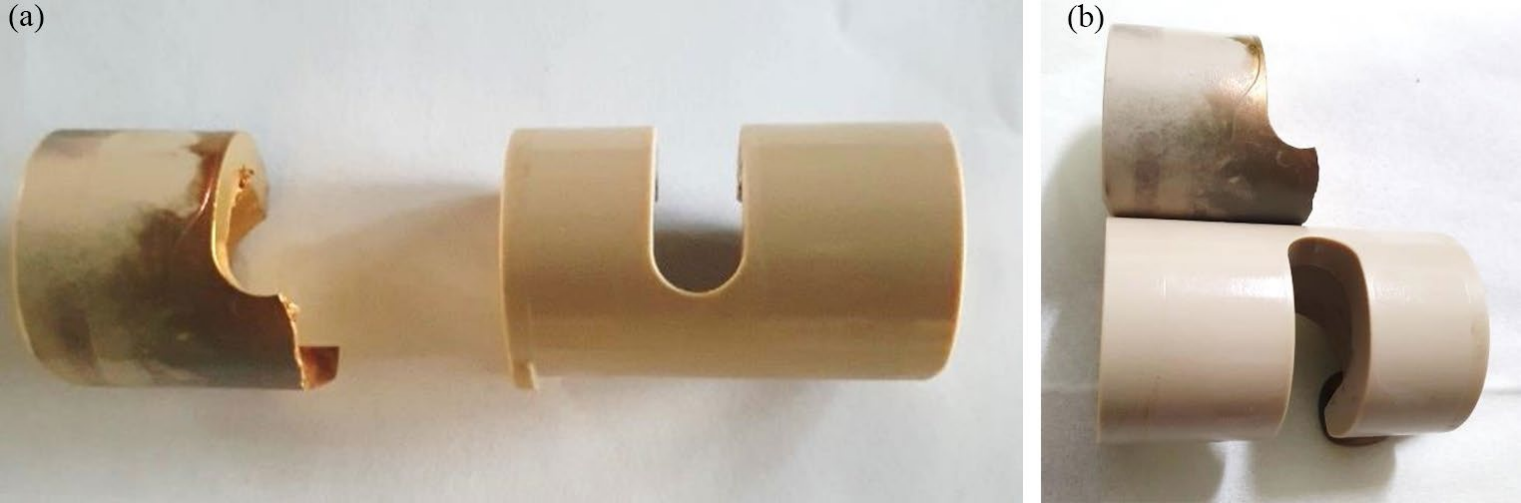
Figure 2a illustrates a side-by-side view of the broken PEEK component compared to the original. Figure 2b illustrates one above the other emphasizing the fracture location as compared to the complete one. The failure occurred at the point of impact during full extension, at the root of the semicircle, where the metal post impacts the PEEK

**Fig. 3:**
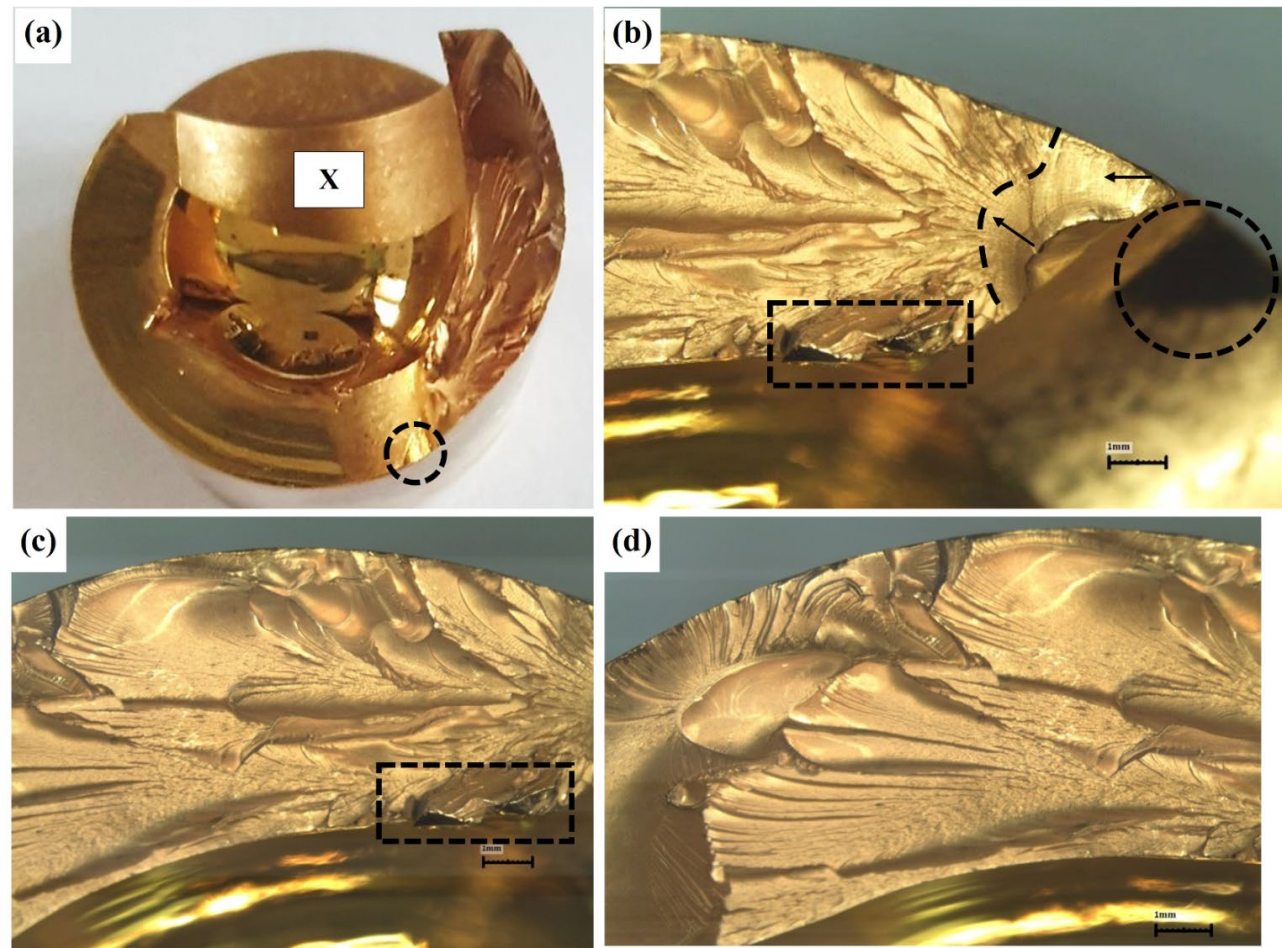
Figure 4a-d depicts the macroscopic failure modes. Two main macroscopic failure modes were noticed divided by the dashed line. The first zone characterized subcritical stable crack growth followed by unstable fast crack propagation

**Fig. 4:**
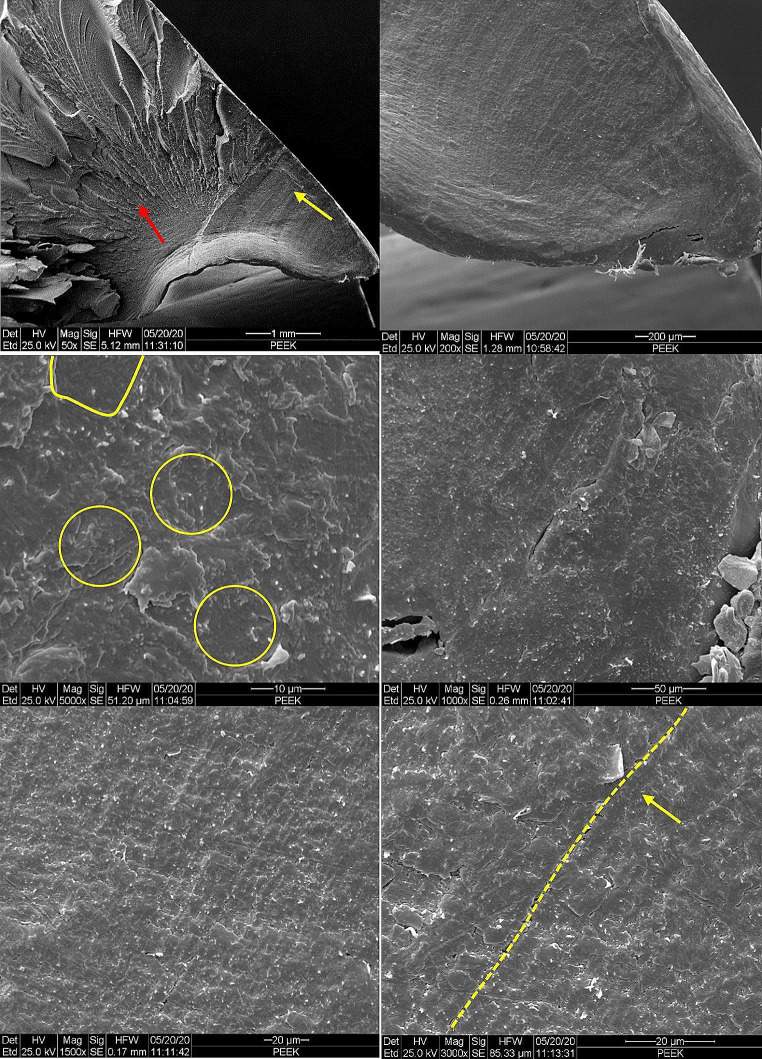
Fracture mode transition during fatigue crack propagation; (**a**) macroscopic fracture mode slow and unstable crack growth, (**b**) flat fatigue mode, (**c**) parabolic markings like, (**d**) flat fracture with some indication of striations like, (**e**) fatigue striations, (**f**) additional alternative mode in the form of micro-cracking

Implant failure analyses of three PEEK lock retrievals (one broken and two worn down) were compared to a new un-used PEEK Optima implant. Figure 1 illustrates the different components of the distal femur prosthesis. The PEEK failure occurred at the stress concentration at the end of the slot as shown in Fig. 2a-b. Failure occurs at the point of maximal extension due to stress concentration from cyclic loading. Figure 3 shows the macroscopic fracture modes. A flat crack is limited by a dashed line followed by a rough one with fracture features that points out the crack propagation direction (Fig. 3b). This is the slow growing subcritical crack growth which occurs initially. The second, with river lines type fracture with coarse features (Fig. 3b-d) representing catastrophic unstable crack propagation. The dashed circle line in Fig. 3a points out the fretting zone which was the source to crack initiation. Figure 4a-b shows scanning electron microscopy images of smooth fracture with the transition from a slow subcritical crack growth to catastrophic failure. Figure 4e-f depicts fatigue striation as observed in metallic materials. Here micro-cracks were detected as the crack velocity increases (see dashed line in Fig. 4f).

**Fig. 5:**
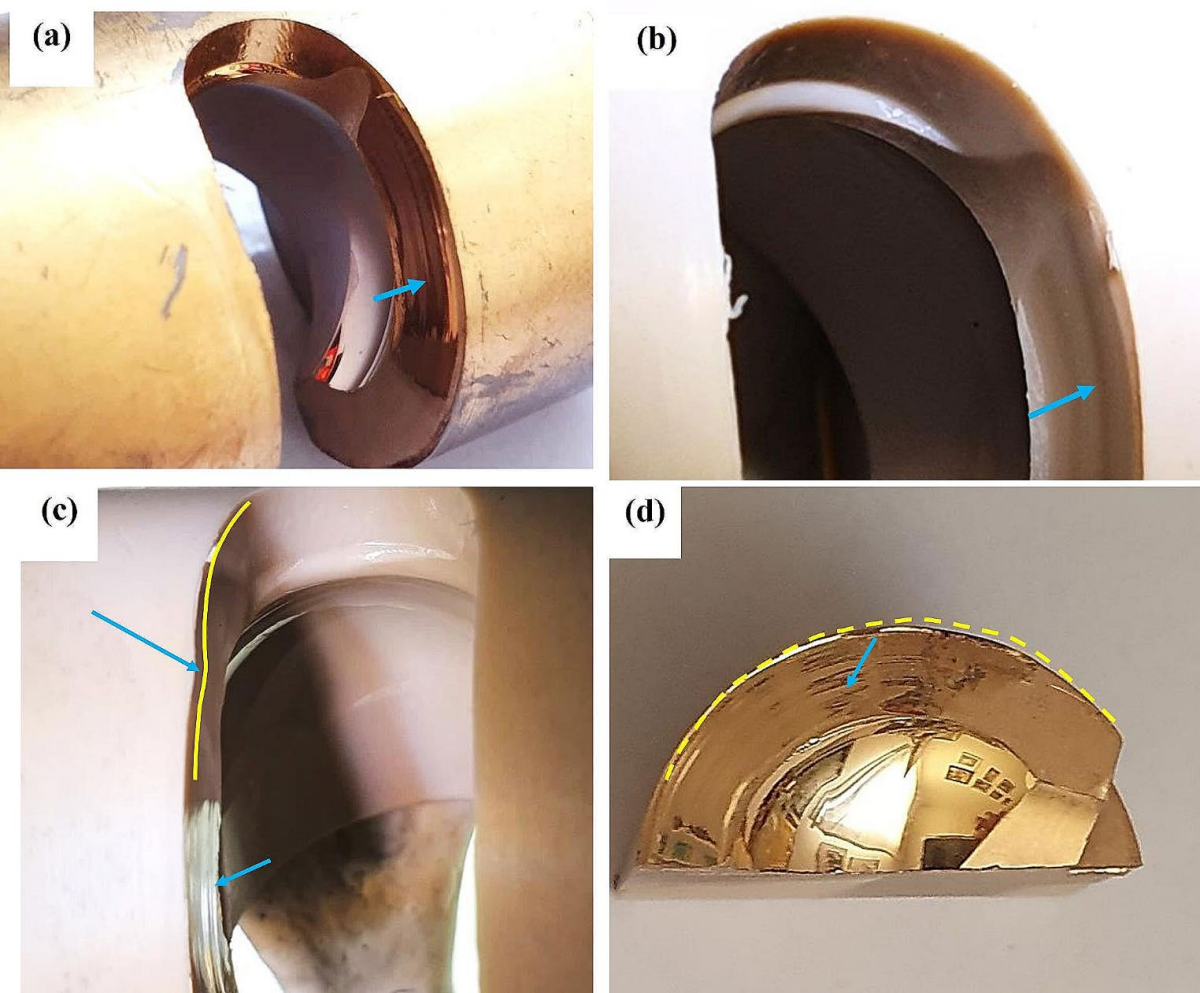
Fretting wear damage on both sides of the slot walls (see arrows in Fig. 5a-d) which affected the symmetry of the slot as marked by dashed line together with an arrow (Fig. 5c). These surfaces are shinier (emphasized by the gold coating needed for the SEM characterization) as compared to the rest of the wall with a brilliant appearance, characterized by circumferential traces. The wear traces which started from the end of slot radius, extended up to 10–15 mm ahead and resulted in the formation of a miniature wall at the outer side (see dashed line in Fig. 5d)

**Fig. 6:**
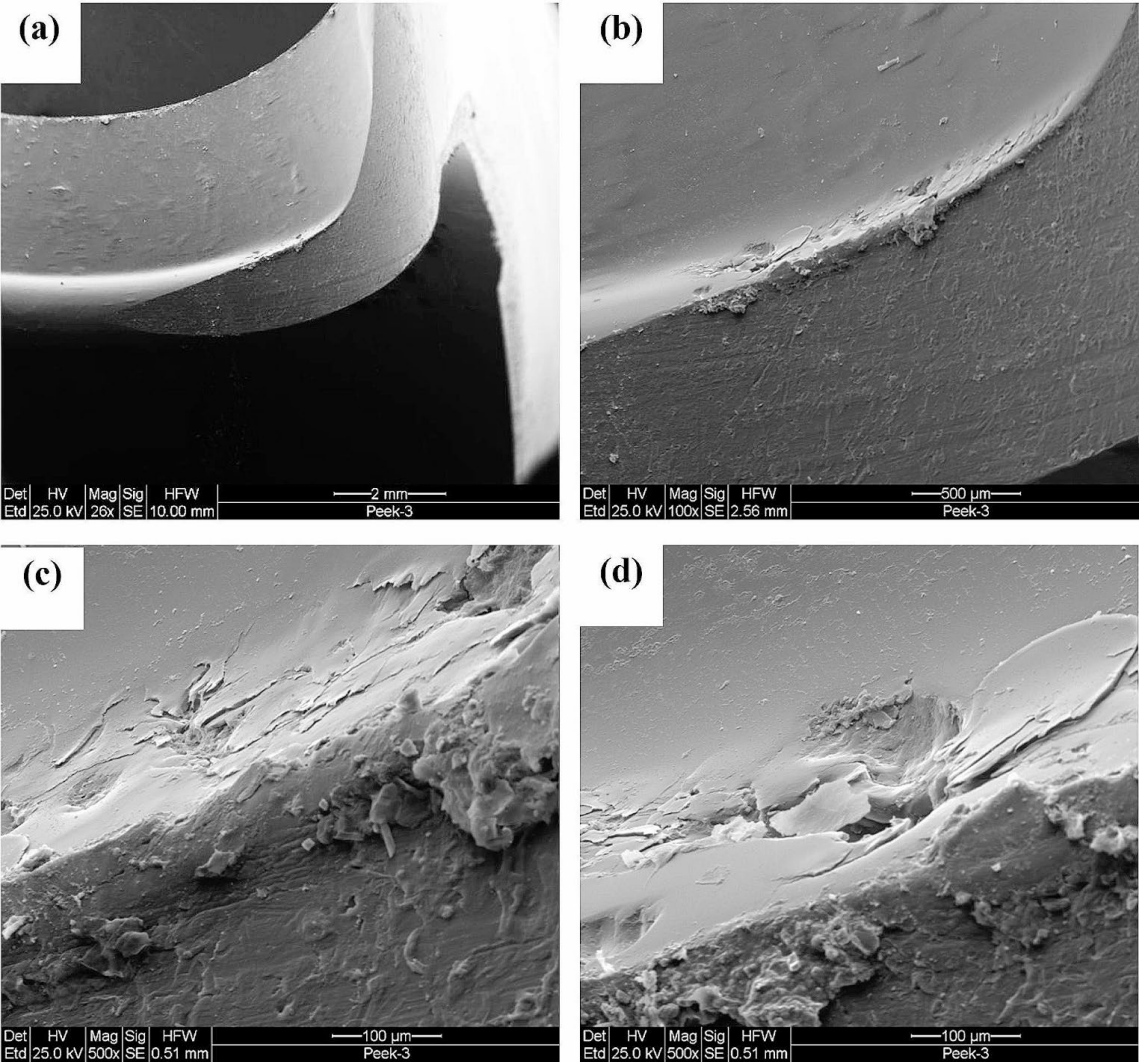
Scanning electron microscope (SEM) tracking on the knoll bottom at the right side (Fig. 6a) indicates that the damage due to the material displacement appears as a surface network of micro-cracks as shown in Fig. 6c-d with different magnifications

**Fig. 7:**
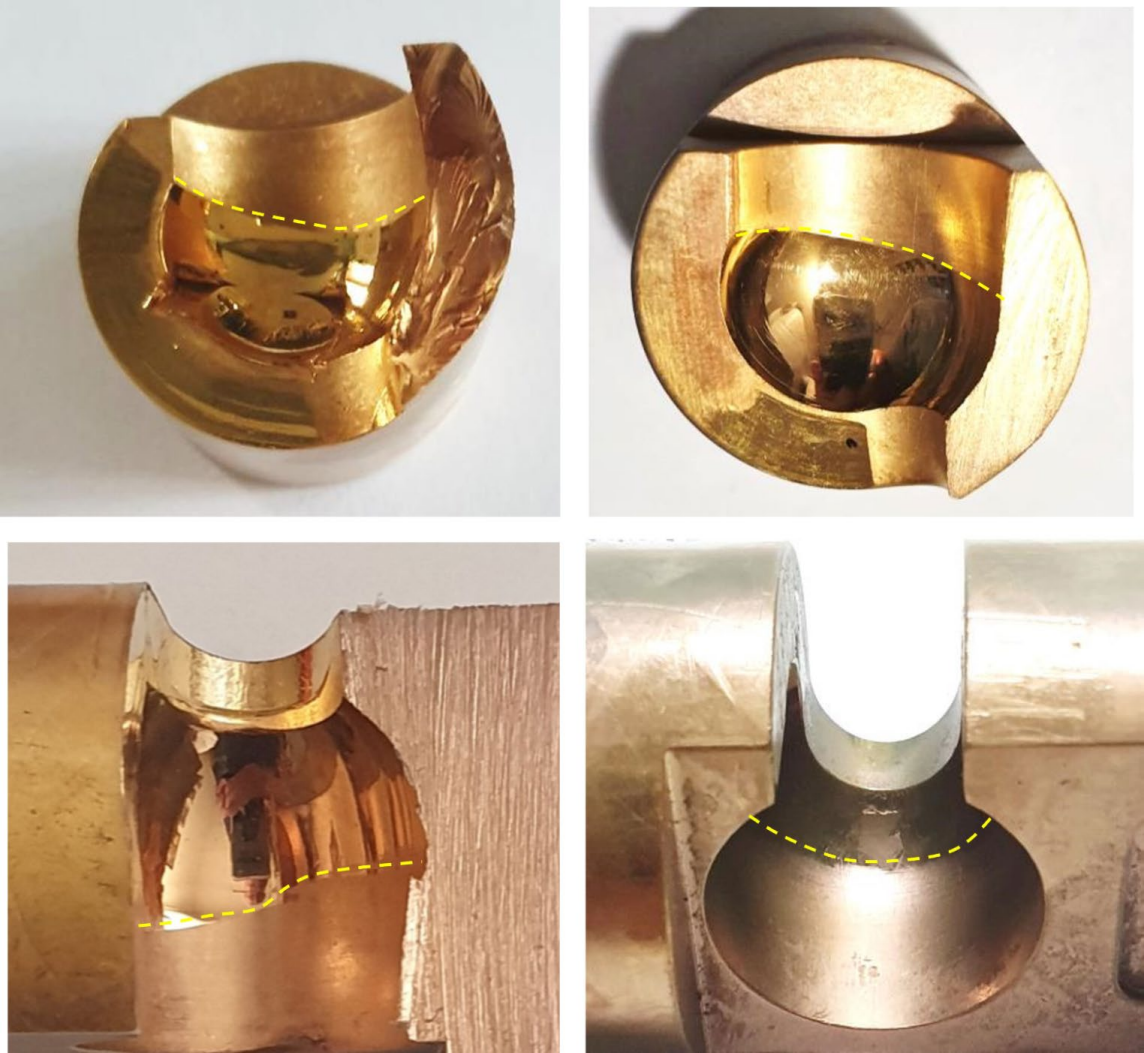
The asymmetrical structure in the semi-spherical as shown in Fig. 7a-b in two separate failed PEEK components. As a result of material displacement due to cyclic loading damage. A third one PEEK shows the same phenomenon with more irregularity as shown in Fig. 7c. This unlikely condition was not detected in the reference PEEK lock as displayed in Fig. 7d. Beside this undesired shape, the transition between the two zones, the shiny and the brilliant ones is accompanied by a small step which can be sensed by passing a finger, a situation which was not found in the reference lock

Failure analysis of the PEEK which did not fail shows significant wear both at the tip of the slot (impacted in full extension) and along the slot sidewalls. (Fig. 5) SEM images (Fig. 6) show this sidewall fretting damage with PEEK material displacement leading to irregularity which leads to joint instability. This was shown to occur in all three failed PEEK retrievals. (Fig. 7)

## Discussion

In our study, we observed a relatively high occurrence of mechanical failures (37.5%) in patients who underwent distal femur reconstruction using Implantcast’s MUTARS® PEEK Optima® system. The majority of patients who experienced prosthesis failure shared a similar clinical pattern, presenting with new onset knee instability several years after surgery, often triggered by activities such as going downstairs. Clinical examination revealed hyperextension of the prosthetic knee, and revision surgery showed a broken or worn-out PEEK-hinge mechanism while the rest of the prosthesis remained intact. To investigate the possible reasons for these failures, we conducted a retrospective analysis of the performance of the PEEK-hinge based prosthesis.

The analysis of mechanical failures involved examining the PEEK-locks that were extracted from the failed implants. Our findings revealed that the failure process initiated with the formation of microcracks due to fretting mechanisms in areas of high stress and strain concentration on the surface of the component. These microcracks gradually propagated under cyclic loading, eventually leading to a complete fracture of the implant. We noted this phenomenon at the tip of the PEEK slot in full extension and on both sidewalls of the slot. One possible explanation for the high failure rate could be attributed to the unique physical properties of PEEK. PEEK has a higher Young’s modulus of elasticity value (4.0 GPa) compared to Ultra High Molecular Weight Polyethylene (UHMWPE) (0.5–1.3), but significantly lower than Titanium alloy Ti-6Al-4 V (116 GPa). This means that PEEK is stiffer than UHMWPE but more brittle and with lower resistance to plastic deformation than Titanium alloy. This inherent brittleness of PEEK may contribute to its higher failure rate compared to UHMWPE. PEEK wear occurred in areas of direct contact with the metal hinge post.

Revision surgery for the failed PEEK component consisted of either replacing only the failed PEEK component, with a new PEEK component, a relatively minor surgical procedure, immediately restoring the implants stability, or, a considerably more extensive revision procedure, replacing the entire prosthesis with the newer generation implantcast MUTARS’s MK, metal on metal, hinged system. Revising the implant to a metal hinge, dictates replacing the whole tibial component, a procedure that carries significant bone loss. The main disadvantage of replacing only the PEEK component proved to be a significant risk for an additional PEEK lock failure. Full scale revision surgery into the newer MK system proved effective in preventing a recurrent mechanical failure, however, increases the risk of common arthroplasty revision surgery complications such as peri-prosthetic joint infection.

There are several limitations to our study, including a relatively small study cohort of 56 patients, and incomplete data on patient weight in 10 cases. Additionally, it is important to note that due to the inherent aggressiveness of bone sarcomas, some patients in our study had relatively short follow-up durations, resulting in limited observation time for outcomes assessment. This limitation may affect the generalizability of our findings and should be considered when interpreting the results.

The failure of the implants in our study could be attributed to multiple factors, including the PEEK material, hinge design, and component production process. However, we were unable to pinpoint a single root cause of failure. Our statistical analysis did reveal a significant association between failure and male gender as well as heavier weight at the time of failure, and the mechanical nature of the failures suggested that the current design of the PEEK hinge joint may not be sufficient and may require changes and improvements to address these issues. Further research and modifications to the PEEK hinge system may be necessary to improve its performance and reduce the occurrence of failures in patients.

## Conclusions

Our findings suggest that PEEK hinge failure in a distal femur reconstruction system is correlated with patient weight and male gender. Retrieval analysis revealed that failure was related to a fretting process between the metallic component and the PEEK which enhanced the crack initiation stage followed by crack propagation due to cyclic loading, leading to instability and mechanical failure of the PEEK component in full extension.

### Electronic supplementary material

Below is the link to the electronic supplementary material.

Supplementary Material 1
